# Contribution of Cyclooxygenase End Products and Oxidative Stress to Intrahepatic Endothelial Dysfunction in Early Non-Alcoholic Fatty Liver Disease

**DOI:** 10.1371/journal.pone.0156650

**Published:** 2016-05-26

**Authors:** Francisco Javier Gonzalez-Paredes, Goretti Hernández Mesa, Dalia Morales Arraez, Raquel Marcelino Reyes, Beatriz Abrante, Felicitas Diaz-Flores, Eduardo Salido, Enrique Quintero, Manuel Hernández-Guerra

**Affiliations:** 1 Institute of Biomedical Technologies and Center of Biomedical Research of the Canary Islands (CIBICAN), University of La Laguna, La Laguna, Tenerife, Spain; 2 Gastroenterology Department, University Hospital of the Canary Islands, La Laguna, Tenerife, Spain; 3 Central Laboratory, University Hospital of the Canary Islands, La Laguna, Tenerife, Spain; 4 Department of Medicine and Psychiatry, University of La Laguna, La Laguna, Tenerife, Spain; IDIBAPS–Hospital Clinic de Barcelona, SPAIN

## Abstract

**Introduction:**

Metabolic syndrome induces endothelial dysfunction, a surrogate marker of cardiovascular disease. In parallel, metabolic syndrome is frequently associated with non-alcoholic fatty liver disease (NAFLD), which may progress to cirrhosis. The aim of the present study was to evaluate intrahepatic endothelial dysfunction related to cyclooxygenase end products and oxidative stress as possible mechanisms involved in the pathophysiology of NAFLD.

**Materials and Methods:**

Sprague-Dawley rats were fed standard diet (control-diet, CD) or high-fat-diet (HFD) for 6 weeks. Metabolic syndrome was assessed by recording arterial pressure, lipids, glycemia and rat body weight. Splanchnic hemodynamics were measured, and endothelial dysfunction was evaluated using concentration-effect curves to acetylcholine. Response was assessed with either vehicle, L-N^G^-Nitroarginine (L-NNA), indomethacin, tempol, or a thromboxane receptor antagonist, SQ 29548. We quantified inflammation, fibrosis, oxidative stress, nitric oxide (NO) bioavailability and thromboxane B_2_ levels.

**Results:**

HFD rats exhibited metabolic syndrome together with the presence of NAFLD. Compared to control-diet livers, HFD livers showed increased hepatic vascular resistance unrelated to inflammation or fibrosis, but with decreased NO activity and increased oxidative stress. Endothelial dysfunction was observed in HFD livers compared with CD rats and improved after cyclooxygenase inhibition or tempol pre-incubation. However, pre-incubation with SQ 29548 did not modify acetylcholine response.

**Conclusions:**

Our study provides evidence that endothelial dysfunction at an early stage of NAFLD is associated with reduced NO bioavailability together with increased cyclooxygenase end products and oxidative stress, which suggests that both pathways are involved in the pathophysiology and may be worth exploring as therapeutic targets to prevent progression of the disease.

## Introduction

Non-alcoholic fatty liver disease (NALFD) is characterized by fat accumulation in the liver that can progress from simple steatosis to steatohepatitis, characterized by hepatocellular injury and varying degrees of fibrosis [1]. NAFLD is considered a major health problem as it is associated with metabolic syndrome, a highly prevalent chronic disease [2]. In addition to increased risk of cardiovascular mortality [3], it is a potentially important cause of liver-related morbidity and mortality, especially in advanced stages with progression to cirrhosis and hepatocellular carcinoma [4].

Molecular mechanisms involved in disease progression in NAFLD are still poorly understood. From a physiopathological point of view, the liver endothelium plays an important homeostatic role in normal relaxation, anti-platelet aggregation, and anti-inflammatory cell adhesion, thus protecting the liver and favoring regeneration after liver injury [5–9]. As in peripheral vessels, nitric oxide (NO) bioavailability in the liver is associated with endothelial function and its reduction contributes to increased hepatic resistance and the development of portal hypertension [10]. This was reported more than a decade ago in cirrhotic livers [5], but recent evidence shows that endothelial dysfunction also occurs early after steatosis induction linked to NO deficiency [11,12]. This is shown by an attenuated response to acetylcholine (ACh) and increased intrahepatic vascular resistance, which is present before any remarkable sign of inflammation or fibrosis. Besides NO, other pathways involved in NAFLD-related endothelial dysfunction have not been extensively explored, but might have therapeutic implications since the pharmacological target could be shifted towards specific pathways in early stages of the disease.

Cardiovascular disease also correlates with metabolic syndrome and several studies indicate that systemic vascular endothelium dysfunction induces progression of vascular disease, such as atherosclerosis [13,14]. Reduced endothelial NO synthase activity, and more recently both decreased NO bioavailability (due to the presence of reactive oxygen species that reacts with NO), and increased cycloxygenase (COX) activity with high production of prostanoids and tromboxane A2 may contribute to vascular dysfunction [15], and have been shown to be critical modulators of vascular tone under normal and pathophysiological circumstances.

Given that endothelial dysfunction is an early event in NAFLD and amenable to treatment that could prevent progression, our aim was to determine, in an animal model of early NAFLD, whether COX end products and oxidative stress are involved in endothelial dysfunction.

## Materials and Methods

### Animals and induction of NAFLD

Male Sprague-Dawley rats weighing 250–300 g were fed during 6 weeks with high fat chow (High fat diet, HFD; Harlam TD.06414, 60% of total calories come from fat, 21% from carbohydrates and 18% from proteins) or standard rat chow (Control diet, CD; Harlan Laboratories, Spain) and provided drinking water *ad libitum*. All rats were maintained on a 12:12-h light-dark cycle and housed in accordance with space recommendations of the National Institutes of Health Guide for the Care and Use of Laboratory Animals (NIH Pub. No. 85–23, Revised 1985) in an animal care facility at the University of La Laguna. All protocols were approved by the Animal Care and Use Committee of the University of La Laguna.

All surgery was performed under anesthesia, and all efforts were made to minimize suffering. On the day of the study, after fasting overnight to ease portal vein cannulation and avoid increased postprandial blood flow, rats were weighed and anesthetized with ketamine (100 mg/kg body weight, Imalgene 1000; Merial, Lyon, France) plus midazolam (5 mg/kg body weight, Laboratorio Reig Jofré, S.A., Barcelona, Spain) before hemodynamic experiments. After hemodynamic studies, animals were sacrificed by exsanguination before extraction of samples. Samples were stored at -80°C.

### Glucose tolerance test

Glycemia was determined in CD (n = 3) and HFD (n = 5) rats with a glucose sensor (Accutrend, Roche, Spain) at baseline, 30, 60 and 120 minutes after administration of a peritoneal glucose bolus (2 g/kg; Sigma-Aldrich, Tres Cantos, Madrid, Spain).

### Analysis of serum biochemical parameters and hepatic triglyceride and cholesterol content

Blood samples (1–2 ml) were collected from the tail of CD (n = 8) and HFD (n = 12) rats and the serum was obtained for the measurement of AST and ALT (DT60/DT60 II, Johnson & Johnson), as well as cholesterol and triglycerides (VITROS 5600 Integrated System, Johnson & Johnson). All the samples were analyzed in the Central Laboratory of the University Hospital of the Canary Islands.

### Western blot analysis

#### Protein nitrotyrosination

Protein nitrotyrosination, a marker of peroxynitrite production and oxidative stress due to NO reaction with reactive oxygen species, was determined by western blotting. Liver tissue from CD and HFD rats (n = 4, in each group) was homogenized, at a ratio of 100 mg tissue per ml RIPA lysis buffer. Protein concentration was assessed by bicinchoninic acid assay, and 50–200 μg protein were denatured in Laemmli’s buffer, separated on 8% SDS-PAGE gels, and transferred to nitrocellulose membranes. Blots were probed with either mouse anti-3-nitrotyrosination monoclonal antibody diluted 1:1000 (Sigma; Madrid, Spain) or mouse anti-glyceraldehyde-3-phosphate dehydrogenase (GAPDH) antibody, diluted 1:1000 (Santa Cruz Biotechnology, Santa Cruz, California, USA). Then, membranes were incubated with HRP-conjugated anti-rabbit IgG (Jackson InmunoResearch Laboratories) or with HRP-conjugated anti-mouse (Jackson InmunoResearch Laboratories), diluted 1:10000. Densitometry of digital images was performed using Melanie v.6 software. GAPDH was used as control of sample loading.

#### p-eNOS

Phosphorylated endothelial NO synthase (p-eNOS) protein detection was performed with rabbit anti-p-eNOS diluted 1/500 (Cell Signaling Technology) as described above. GAPDH was used as control of sample loading.

### Myeloperoxidase activity

Myeloperoxidase (MPO) activity was measured in CD (n = 6) and HFD (n = 11) rats as previously described, with some modifications [16]. In brief, 100 mg of liver tissue were homogenized in 1 ml of 50 mM potassium phosphate buffer (pH 6) containing 0.5% (wt/vol) hexadecyl-trimethyl-ammonium-bromide. Homogenates were incubated with 0.167 mg/ml O-dianisidine (Sigma-Aldrich, Madrid, Spain) and 0.3% (vol/vol) H_2_O_2_ (Sigma-Aldrich, Tres Cantos, Madrid, Spain). After an incubation period of 5 min at 25°C, absorption at 450 nm was measured. Homogenates protein concentrations were quantified by bicinchoninic acid assay. Results were expressed in units of MPO activity per gram of wet tissue (uMPO/g).

### *In vivo* hemodynamic studies

Rats (n = 16) were anaesthetized as previously mentioned. A tracheotomy and cannulation with a catheter made of polyethylene tubing (PE-240, Portex, Kent, UK) was performed in order to maintain adequate respiration during the anesthesia. Then, a PE-50 catheter was inserted in the carotid artery to monitor blood pressure (mmHg) to estimate mean arterial pressure (MAP) from systolic (SAP) and diastolic (DAP) arterial pressure, and into the portal vein through an ileocolic vein to measure portal pressure (mmHg). Perivascular ultrasonic transit-time flow probe (2PR, 2 mm, Transonic System, Ithaca, NY, USA) connected to a flow meter (T201, Transonic System, Ithaca, NY, USA) were placed around the portal vein, as close as possible to the liver to measure portal blood flow (mL/min/g liver). Hepatic vascular resistance was calculated as portal pressure/portal blood flow (mmHg/mL/min/g liver). The catheters were connected to a Power Lab (4SP) linked to a computer using the Chart V5.5.6 for Windows software (ADInstruments, Mountain View, CA, USA). The temperature of the animals was maintained at 37°± 0.5°C and after a period of 10 minutes of stabilization, recordings were performed.

### Evaluation of intrahepatic endothelial dysfunction

#### Isolated perfused liver system

Rats (n = 39) were anaesthetized as previously described. A flow-controlled perfusion system was used in this study. The system has been described elsewhere [17]. The perfused rat liver preparation was allowed to stabilize for 20 minutes before the vasoactive substances were added. The criteria used to determine liver viability were gross appearance, stable perfusion, bile production (over 0.4 ml/min per gram of liver) and a stable buffer pH (7.4±0.1) during the stabilization period. If one of the viability criteria was not met, the experiment was discarded.

#### Portal perfusion pressure dose-response curve to acetylcholine

Baseline perfusion portal pressure was recorded before the intrahepatic microcirculation was pre-constricted with the alpha-1-adrenergic agonist methoxamine (Mtx, 10^-4^M), a well-characterized vasoconstrictor of hepatic vasculature. Five minutes later, dose-response curves to cumulative doses of acetylcholine (ACh, 10^−8^, 10^−7^ and 10^-6^M) were evaluated. The concentration of ACh was increased by one log unit every 1.5 min. Response to cumulative doses of ACh was calculated as a percent change in PP.

#### Assessment of endothelial dysfunction related to NO, oxidative stress, COX and thromboxane pathways

In subgroups of HFD and CD animals, endothelial dysfunction was assessed after preincubation with either vehicle (n = 17), L-NNA (n = 9, N^W^-nitro-L-arginine NO synthase inhibitor; 10^−3^ M), indomethacin (n = 5, nonselective COX inhibitor; 20 μM), tempol (n = 4, 4-Hydroxy-TEMPO, oxidative stress scavenger; 10^−3^ M) or SQ 29548 (n = 4, blocker of the common receptor for PGH2 and thromboxane A_2_, 1 μM) [18].

### Nitric oxide bioavailability

Briefly, equal amounts of liver tissue (200 mg) from CD and HFD rats (n = 4, in each group) were dropped into 10 volumes of 5% trichloroacetic acid and homogenized at 4°C. The precipitate was removed by centrifugation and the supernatant was washed with water-saturated diethyl ether, and lyophilized. Extracts were dissolved in assay buffer, and GMPc levels were determined by enzyme immunoassay (Cayman Chemical Co., Ann Arbor, MI).

### Hepatic lipid peroxidation analysis

In brief, rat liver pieces (n = 5, in each group) were weighed exactly (between 100–150 mg of tissue) and homogenized in 1 ml of ice-cold saline solution. 0.2 ml aliquots of homogenate were used for malondialdehyde (MDA) determination. MDA levels, referred to as thiobarbituric acid-reactive substance, were measured according to a previously described method [19]. The pink complex of samples was extracted in n-butanol. Each one was placed in a 96 well plate and read at 535 nm in a microplate spectrophotometer reader (Spectra MAX-190, Molecular Devices, Sunnyvale, CA 94089, USA). The detection limit of this assay was 0.079 nmol/ml; the intra- and inter-assay CV were 1.82% and 4.01%, respectively. The sample concentration of MDA was expressed in nmol/g of hepatic wet tissue.

### Thromboxane B_2_ quantification

To determine if indomethacin correctly blocked the production of cyclooxygenase end products, in a subgroup of rats (n = 4, in each group) samples of perfusate (2 mL) of HFD livers preincubated with indomethacin or vehicle were obtained before and after administration of Mtx/ACh. Thromboxane B_2_ (the end metabolite of thromboxane A_2_) was quantified in duplicate in perfusate samples using the commercial kit “Thromboxane B_2_ EIA Kit” (Cayman Chemical Co., Ann Harbor, MI). Thromboxane B_2_ production was expressed as total increase/decrease over baseline after Mtx/ACh administration.

### Histopathology

Liver tissues from CD (n = 4) and HFD rats (n = 6) were fixed in 10% formalin, embedded in paraffin, sectioned, and stained with hematoxylin-eosin and sirius-red. In addition, anti-adipophilin mouse monoclonal antibody diluted 1/100 was used to identify cytoplasmic lipid vesicles (Acris Antibodies, Hiddenhausen, Germany). Labelled polimer-HRP EnVision^TM^ Dual Link System-HPR (Dako, Denmark) was used to visualize staining for adipophilin. Six fields from each slide were randomly selected, photographed, and analyzed using a microscope equipped with a digital camera (Olympus, Tokyo, Japan) at x20 to x40. Hematoxylin-eosin preparations were used to determinate lobular inflammatory activity and steatosis, in addition to immunohistochemistry staining for adipophilin. Score for inflammation was performed as previously described [20]: 1) focal collections of mononuclear inflammatory cells, 2) diffuse infiltrates of mononuclear inflammatory cells, 3) focal collections of polymorphonuclear cells in addition to mononuclear cell infiltrates, or 4) diffuse infiltrates of polymorphonuclear cells in the parenchymal area. Steatosis was scored as previously described [21]: 0) steatosis in <5% of the hepatocytes,1) 5–33% of hepatocytes, 2 >33–66% of hepatocytes, and 3) >66% of hepatocytes. Sirius-red stained slides were used for semiquantitative analysis of hepatic fibrosis. Positive stained areas were quantified using ImageJ software (http://rsb.info.nih.gov/ij) and values expressed as arbitrary units.

### Hepatic hydroxyproline quantification

Hepatic hydroxyproline content was measured in 100 mg liver tissue (n = 5 in each group) using the commercial kit “Hydroxyproline Assay Kit” (Sigma-Aldrich,Tres Cantos, Madrid, Spain) following the manufacturer's instructions. Results were expressed as micrograms of hydroxyproline per gram of wet tissue (μg hydroxyproline/g).

### Drugs and reagents

Mtx, ACh, tempol, L-NNA and indomethacin were purchased from Sigma-Aldrich (Tres Cantos, Madrid, Spain). SQ 29548 was obtained from Cayman Chemical Co. (Ann Harbor, MI).

### Statistical analysis

Statistical analysis was performed using SPSS 15.0 for Windows (SPSS Inc., Chicago, IL). Comparisons between groups were performed using analysis of variance (ANOVA) followed by Student´s t-test or the non-parametric test for unpaired data (Mann-Whitney) as appropriate. All data are reported as means±SEM values. Differences with a p value <0.05 were considered significant.

## Results

### Assessing metabolic syndrome

#### Body and liver weight

**Fig 1:**
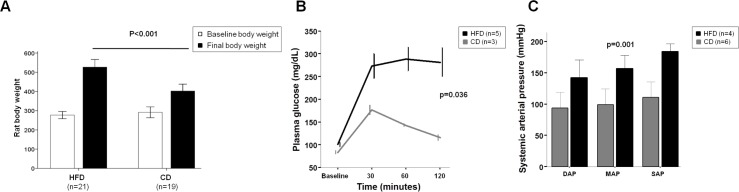
Metabolic syndrome assessment. (A) Rat body weight. Rats fed with HFD (n = 21) increased rat body weight compared with CD rats (n = 19). (B) Glucose intolerance. HFD (n = 5) rats exhibited higher fasting hyperglycemia and at every time point after glucose administration compared with CD (n = 3) rats. (C) Arterial hypertension. Higher mean arterial pressure (MAP), diastolic arterial pressure (DAP) and systolic arterial pressure (SAP) were observed in HFD (n = 4) rats as compared with CD rats (n = 6). Values are mean ± SEM.

Compared with CD rats, those on HFD showed significantly higher final rat body weight (527±64 vs. 402±60 g; p<0.001; Fig 1A). The rat body weight gain was evident in terms of percentage (92.6±35.7 vs. 39.4±19%; p<0.001) and absolute rat body weight increase (249.7±72.5 vs. 110.5±50.2 g; p<0.001). In addition, HFD rats showed higher liver weight compared with CD rats (15.03±2.56 vs. 12.91±2.14 g; p<0.01).

#### Glucose tolerance test

HFD rats exhibited higher fasting hyperglycemia levels compared with CD rats (101±10 vs. 83±5 mg/dL; p = 0.015). After glucose administration, although this manoeuvre induced hyperglycemia in all groups, a higher level at every time point was observed in HFD rats compared with CD rats (Fig 1B).

#### Arterial pressure

As shown in Fig 1C, there was a significant difference in arterial pressure between HFD and CD rats (Mean arterial pressure: 156±7 vs. 99±10mmHg; p = 0.003).

### Liver steatosis, inflammation and fibrosis

#### Steatosis and inflammation

**Fig 2:**
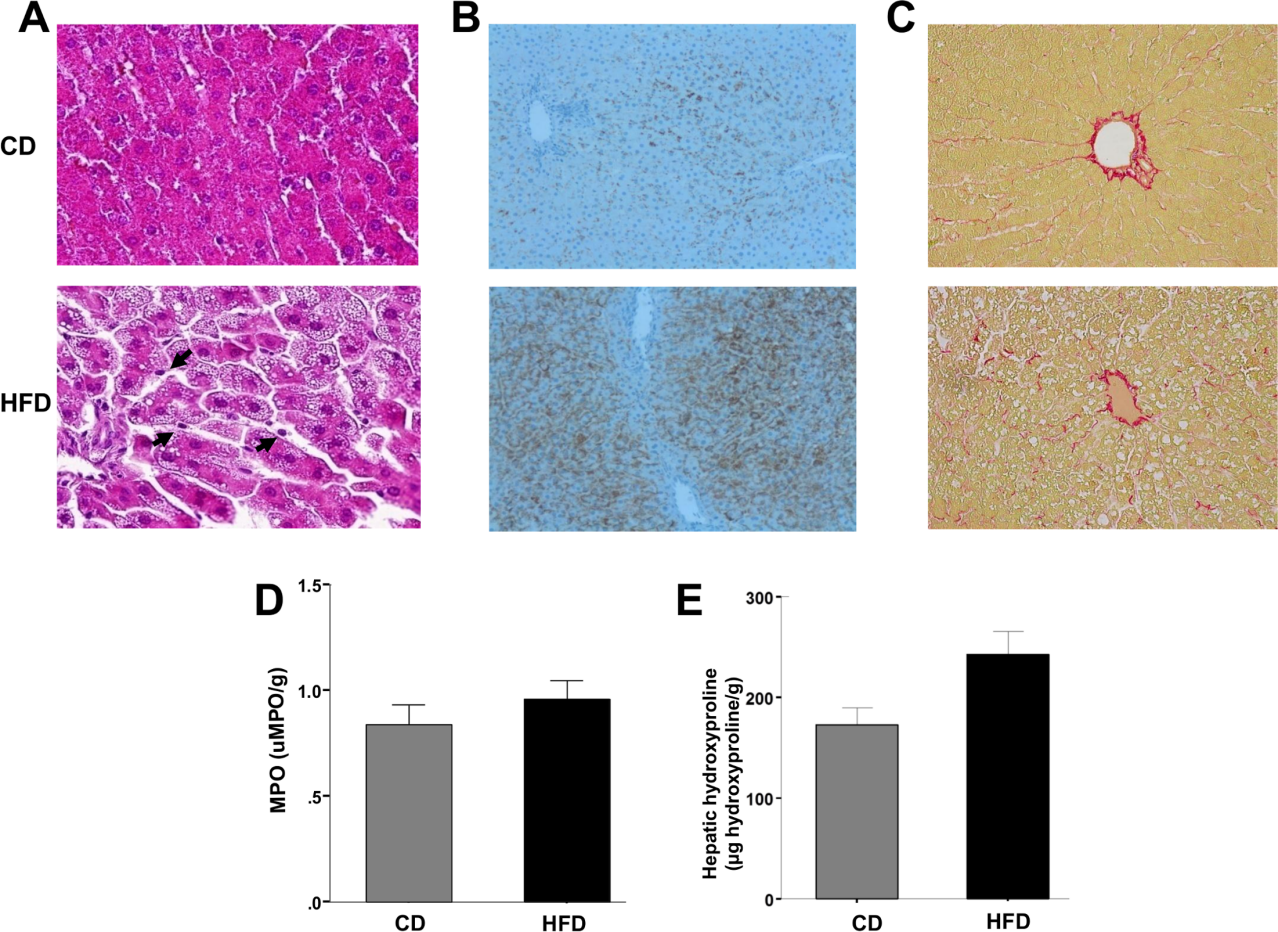
Liver steatosis, inflammation and fibrosis evaluation. (A) Representative histological images of liver tissue stained with hematoxilin-eosin (x40) of CD (*Top*) and HFD (*Bottom*) rats. Livers of HFD rats showed significant microvesicular steatosis, compared with CD rats. Few inflammatory cells were observed (Arrows). (B) Liver slices (x20) showing high content of adipophilin in HFD liver rats (*Bottom*) as compared with CD rats (*Top*). (C) Sirius-red staining (x20) of CD (*Top*) and HFD (*Bottom*) rats. Similar grade of fibrosis was observed. (D) Evaluation of myeloperoxidase (MPO) activity. A similar content of MPO was observed in CD (n = 6) and HFD (n = 11) rats. (E) Evaluation of hepatic hydroxyproline content. A similar content of hydroxyproline was observed in CD and HFD rats (n = 5, in each group). Values are mean ± SEM.

HFD rats showed higher lipid levels in liver tissue, and the opposite was observed in blood samples (Table 1). Myeloperoxidase tissue quantification showed no significant differences between CD and HFD rats (0.84±0.19 vs. 0.98±0.11 uMPO/g, p = 0.545, Fig 2D). AST and ALT levels of CD and HFD rats are shown in Table 1.

**Table 1:** Characteristics of rats receiving control diet (CD) and high-fat diet (HFD).

	CD (n = 8)	HFD (n = 12)	*p*-value
AST (IU)	112±7	94±4	0.028
ALT (IU)	49±2	51±2	0.546
Serum cholesterol (mg/dL)	92±6	69±2	0.001
Serum triglycerides (mg/dL)	184±31	36±3	<0.001
Liver cholesterol (mg/g)	1.00±0.02	1.24±0.06	0.005
Liver triglycerides (mg/g)	3.44±0.18	5.98±0.42	<0.001

AST, aspartate transaminase; ALT, alanine transaminase.

#### Hematoxilin-eosin staining and adipophilin immunohistochemistry

As expected, hematoxilin-eosin staining and adipophilin immunohistochemistry showed more hepatocyte lipid content in HFD livers than in CD livers, scoring at least 2 points (steatosis in >33–66% of the hepatocytes; Fig 2A and 2B). However, in HFD rats we observed few inflammatory cells (Fig 2A, arrows), without significant focal collections.

#### Sirius-red staining

Sirius-red staining showed no differences in hepatic fibrosis between CD and HFD livers (Fig 2C) in histological liver samples after semiquantitative analysis (2.26±0.30 vs. 2.38±0.09 arbitrary units, p = 0.695).

#### Hydroxyproline

Quantification of hepatic hydroxyproline showed no significant differences between the CD and HFD groups (172.7±21.8 vs. 242.6±31.3 μg hydroxyproline/g, p = 0.128, Fig 2E).

### Oxidative stress, p-eNOS and NO bioavailability

**Fig 3:**
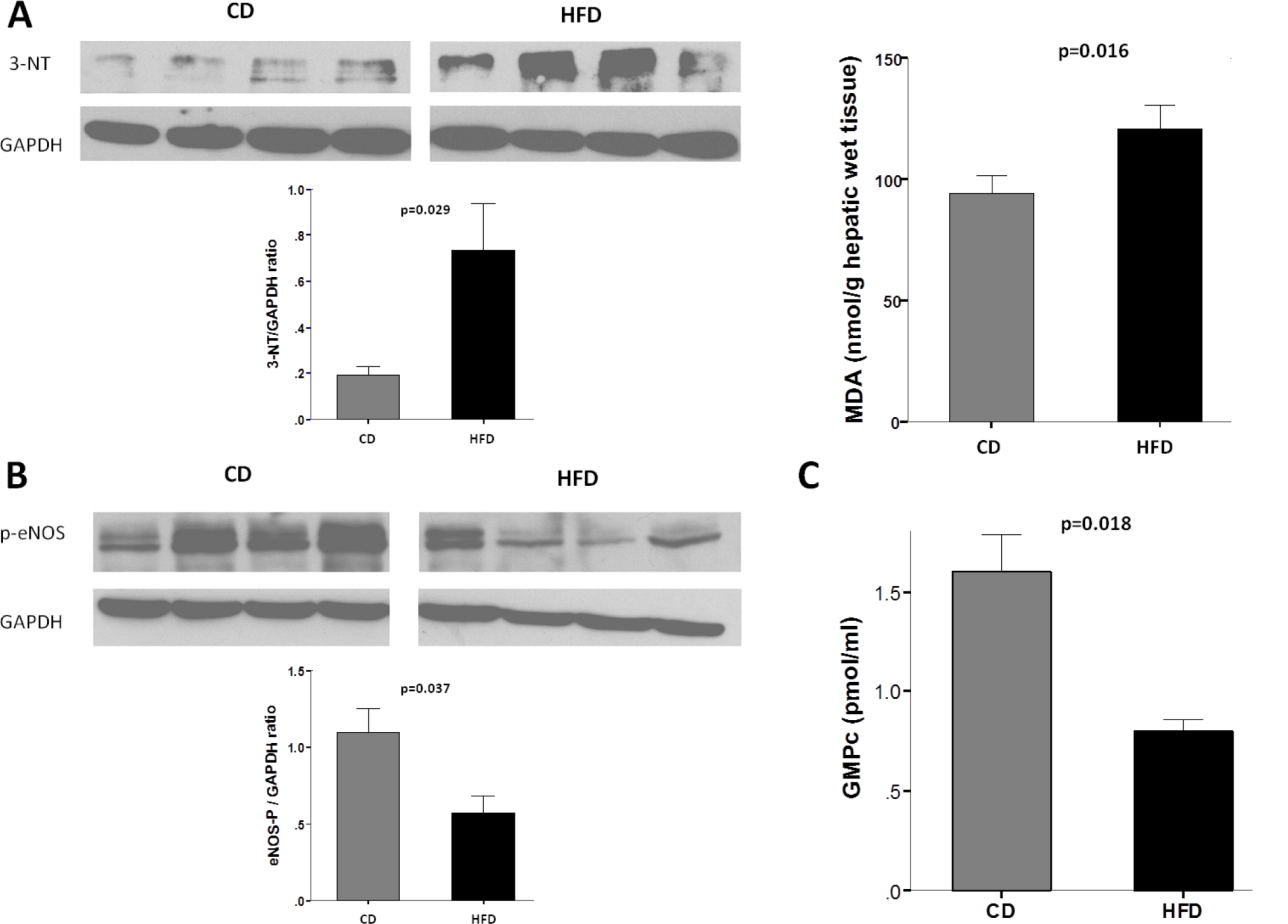
Oxidative stress and nitric oxide (NO). (A) *Left panel*: *Top*. Representative western blot of nitrotyrosinated proteins (3-NT) of livers from HFD and CD rats (n = 4, in each group). *Bottom*. Densitometry analysis of 3-NT to glyceraldehyde-3-phosphate dehydrogenase (GAPDH) ratio. HFD rats exhibited a higher level of oxidative stress. *Right panel*: Malondialdehyde (MDA) content in HFD and CD rats (n = 5, in each group). (B) *Top*. Representative western blot of phosphorylated endothelial NO synthase (p-eNOS) of livers from HFD and CD rats (n = 4, in each group). *Bottom*. Densitometry analysis of p-eNOS to GAPDH ratio. eNOS activity was decreased in HFD rats. (C) NO bioavailability. GMPc intrahepatic levels were significantly different between CD and HFD rats (n = 4, in each group). Values are mean ± SEM.

HFD livers showed increased oxidative stress compared with CD livers, as indicated by higher 3-nitrotyrosination values (Fig 3A, left panel). Furthermore, MDA quantification showed higher content of MDA in HFD livers compared to CD livers (120.88±21.38 vs. 94.41±15.22 nmol/g wet tissue, p = 0.016; Fig 3A, right panel). In parallel, HFD livers showed lower p-eNOS values (Fig 3B). As a result, hepatic GMPc levels were significantly lower in HFD rats compared with CD rats (0.80±0.07 vs. 1.60±0.23 pmol/mL, p = 0.018; Fig 3C).

### Portal and splanchnic hemodynamic study

**Fig 4:**
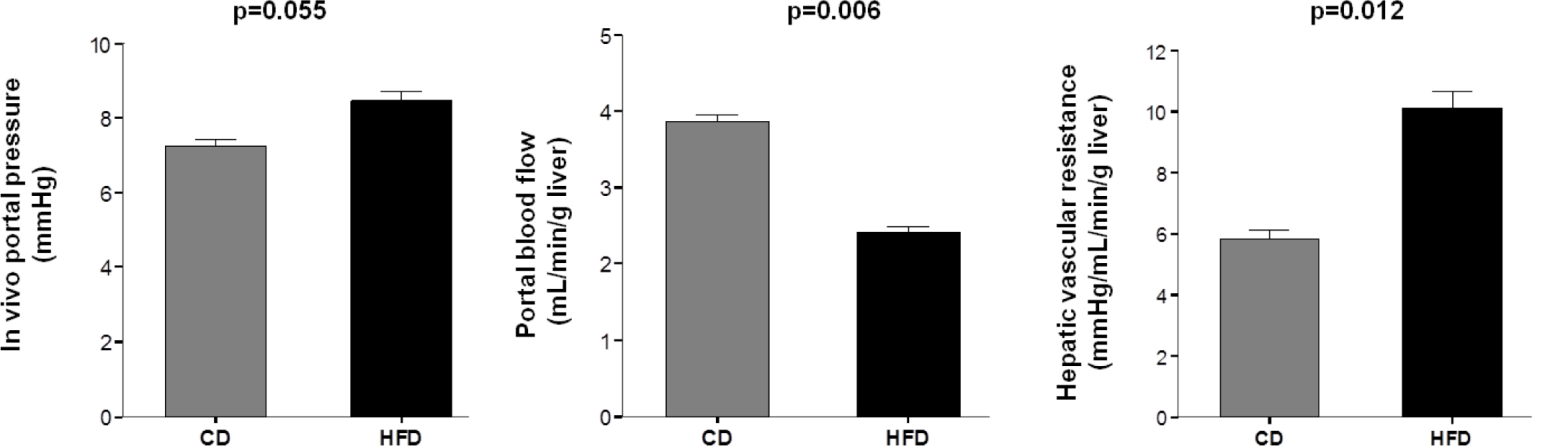
In vivo assessment of liver circulation. Portal pressure was increased in HFD rats (n = 7) compared with CD rats (n = 9). This increase was associated with a significant change in portal blood flow reflecting an increase in hepatic vascular resistance. Values are mean ± SEM.

Portal perfusion pressure in HFD rats was slightly but significantly increased as compared with CD rats, (6.3±0.5 vs. 5.0±0.1 mmHg, p = 0.026). Similarly, HFD rats exhibited higher *in vivo* portal pressure associated to decreased portal blood flow, implying higher hepatic vascular resistance (Fig 4).

### Evaluation of endothelial dysfunction

#### Role of NO

**Fig 5:**
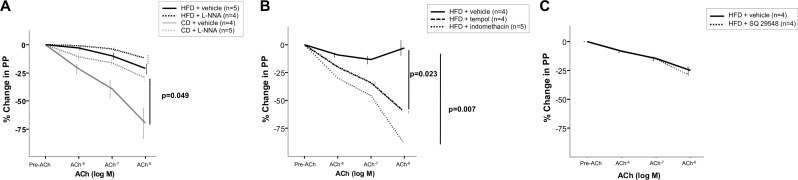
Isolated perfused liver studies. (A) Endothelium-dependent relaxation to acetylcholine (ACh) after preincubation with N^W^-nitro-L-arginine (L-NNA) or vehicle in CD and HFD rats. Vasodilation in CD rats (n = 5) was significantly reduced by endothelial NO synthase inhibition compared with vehicle (n = 4). Conversely, in HFD rats relaxation to ACh was unaffected by L-NNA (n = 4) compared with vehicle (n = 5). (B) Cyclooxygenase blockade and antioxidant preincubation improved vasodilator response in NAFLD rats. HFD preincubated with indomethacin (n = 5) or tempol (n = 4), improved endothelial dysfunction compared with vehicle (n = 4). (C) Effect of SQ29548 on the concentration-response curve to ACh in rats with NAFLD. Tromboxane A2 receptor selective antagonist (n = 4) did not improve vasodilation compared with vehicle (n = 4).

CD livers showed incremental vasorelaxation in response to cumulative doses of ACh, whereas HFD livers showed less vasodilation compared with controls (Maximal at ACh 10^-6^M: -69.7±26.4 vs. -21.1±9.5% in controls; p<0.01, Fig 5A). In CD rats, vasorelaxation in response to ACh was significantly reduced by NO synthase inhibition with L-NNA (Maximal at ACh 10^-6^M: -69.7±26.5 vs. -29.4±11% in L-NNA group; p<0.05), whereas in HFD rats L-NNA response was not different compared with vehicle (Fig 5A).

#### Role of oxidative stress and COX pathway in endothelial dysfunction in HFD rats

In HFD livers, preincubation with tempol or indomethacin compared with vehicle markedly increased the vasorelaxation in response to cumulative doses of ACh (Maximal at ACh 10^-6^M: -60.5±4.4% in tempol group vs. -89.2±9.5% in indomethacin vs. -2.8±14.3% in vehicle group; p<0.05; Fig 5B). However, preincubation with selective thromboxane A_2_ receptor antagonist SQ 29,548 showed no significant changes in vasodilation in response to cumulative doses of ACh (Fig 5C).

As expected, thromboxane B_2_ production was significantly reduced in HFD rats after indomethacin preincubation as compared with vehicle, expressed as absolute change (-29.5+19.5 vs. 108.9+28.9 pg/mL; p = 0.029) or percentage change (-22.1+14.2 vs. 61.8+17.2%; p = 0.029).

## Discussion

The present study provides evidence that COX-derived prostanoids and oxidative stress are important mediators of endothelial dysfunction in an experimental model of NAFLD. In addition, we demonstrate that this occurs at an early stage of NAFLD.

We used a well-described rat model of NAFLD based on chronic over-nutrition. This animal model reproduces the early stage of liver disease characterized by steatosis with low inflammation and no fibrosis, although with features of metabolic syndrome such as overweight, arterial hypertension and insulin resistance [12,22]. From a methodological point of view, this is of paramount importance, since the pathogenic mechanisms responsible for the transition from steatosis to steatohepatitis and fibrosis in early stage NAFLD could be a potential therapeutic target.

Hypertension, dyslipidemia and insulin resistance are major factors contributing to the development of cardiovascular disease. In general, functional alterations due to endothelial dysfunction, characterized by impaired vasodilation, occur early before structural alterations, and constitute a well-known prognostic factor for cardiovascular events [14]. Factors that contribute to vascular dysfunction include reduced endothelial NO synthase activity [20], and more recently both decreased NO bioavailability (due to the presence of reactive oxygen species that scavenge NO) and increased COX activity with vasoconstrictor prostaglandins [21].

To our knowledge, the present study is the first to evaluate the role of COX end products and oxidative stress as pathophysiologic contributors to intrahepatic endothelial dysfunction in a NAFLD model.

Both inflammation events and fibrosis were reduced or negligible in our HFD model of NAFLD, although hemodynamic changes did occur. These take place due to accumulation of lipids and probably swelling of hepatic parenchymal cells together with incipient perivascular fibrosis inducing sinusoidal narrowing, which may, at least partially, explain the observed increase in hepatic vascular resistance [23]. Additionally, many hemodynamic changes are also due to endothelial dysfunction of liver microcirculation. In accordance with previous studies, NAFLD rats showed attenuated intrahepatic vasodilatory response to Ach [12]. Indeed, our functional study clearly showed reduced NO bioavailability in HFD livers, since when livers were preincubated with the NO-inhibitor L-NNA, vasorelaxation in response to ACh was unaffected, whereas vasodilation was attenuated in control livers. In consonance with these results, decreased levels of p-eNOS and GMPc were found in our rat model of NAFLD.

It has been suggested that much of the impaired endothelial function in response to ACh is NO-mediated in NAFLD [24], but the novel finding of our study is that COX end products also play a role in the endothelial-dependent vasodilation of NAFLD livers. Prostanoids are produced from arachidonic acid released from the phospholipids of the sinusoidal cell membranes by COX, thus generating prostaglandin G_2_ and prostaglandin H_2_ [25]. Thus the production of different prostanoids and vascular effects depend on the different types of synthases and receptors, respectively. Of particular interest is thromboxane receptor which has an important role in vasoconstriction after activation by thromboxane A_2_, but other prostaglandins, isoprostanes and hydroxyeicosatetraenoic acids are also involved [26]. As previously shown by others, in vascular disorders such us hypertension [27], diabetes [28], pulmonary hypertension [29], obesity [30] and metabolic syndrome [31], endothelial dysfunction could be due to COX-derived prostanoids and particularly, thromboxane-receptor-mediated vasoconstriction. Indeed, the blockade of COX activity in vessels has been shown to improve endothelial dysfunction [32].

In the liver, similar effects with indomethacin have been observed in cirrhotic rats [33–36] or after specifically blocking the thromboxane pathway [37,38]. Indeed, increased hepatic vascular tone is partly due to increased production of COX-derived vasoconstrictive prostanoids, such as thromboxane A_2_. In our experimental model of NAFLD, indomethacin preincubation prevented thromboxane B_2_ increase after ACh administration and restored vasodilation in HFD livers almost completely. However, preincubation and blockade of prostaglandin H_2_-thromboxane A_2_ receptors with SQ 29548 had no effect on vasodilation, suggesting that other products of COX such as prostaglandin F_2α_ or prostaglandin E_2_ may be involved in endothelial dysfunction in our model of NAFLD [25,39]. Recently, in an experimental model of NAFLD with induced methionine-choline-deficient diet, thromboxane A_2_ synthase expression was shown to be upregulated whereas no alterations in NO were observed. However, the study lacked functional experiments [40]. Our study using specific thromboxane receptor blockers suggests it does not have a role in intrahepatic vasodilation, at least at this early stage of NAFLD.

To date, there is strong supportive evidence that oxidative stress is critically involved in impaired vasodilator responses in diseases such as hypertension and other vascular disorders such as diabetes [41,42]. One of the best-characterized mechanisms whereby reactive oxygen species alter vasodilation is its effect on the NO pathway. Briefly, NO reacts with superoxide anion to produce peroxynitrite thus leading to the loss of vasodilator effects of NO. On the other hand, reactive oxygen species such as superoxide anion and hydrogen peroxide are increased in human and experimental NAFLD [43,44]. Indeed, in our setting, protein nitrotyrosination, a marker of proxynitrite production and oxidative stress, and MDA increased in NAFLD rats compared with controls. Along with the increase in oxidative stress, the endothelium-dependent relaxation was impaired and was unaffected by inhibition of endothelial NO synthase, indicating reduced bioavailability of NO. Moreover, endothelial dysfunction was improved by pretreatment with an antioxidant, suggesting a prominent role for reactive oxygen species in decreased NO bioavailability in NAFLD. Our results are consistent with the findings of experimental studies in cirrhotic rats, and confirm the fact that NO bioavailability is also reduced in NAFLD [45]. These considerations further emphasize that antioxidant therapy at early stages may be useful to improve endothelial dysfunction, before the course is shifted towards proinflammatory and profibrogenic pathways.

Besides the direct interaction of reactive oxygen species with NO, oxidative stress may also modulate COX and COX-derived prostanoid synthases. This has been found in peripheral vessels in hypertension [15] and more recently in cirrhotic livers [46], contributing to increased vasoconstriction. Conversely, reactive oxygen species can derive directly from COX, so theoretically, by blocking the latter more NO is expected to be available [25]. Indeed, this occurred in cirrhotic livers when COX activation with arachidonic acid dramatically increased superoxide levels reducing the already low NO, whereas were prevented by COX inhibition which in turn increased NO bioavailability [46]. Further studies are needed to specifically address this issue, in NAFLD. Nevertheless, the two strategies do not seem to be incompatible and pharmacological manipulation may be beneficial to prevent intrahepatic endothelial dysfunction.

Importantly, chronic treatment with non-steroidal anti-inflammatory drugs (NSAIDs) produces multisystemic adverse effects [47]. These deleterious effects may be due to simultaneous suppression of prostanoids with a vasodilator profile (prostacyclin) and concomitant inhibition of vasoconstrictors [48]. Therefore, drugs targeting downstream COX synthases with a vasoconstrictor profile may represent an effective therapeutic alternative. Alternatively, prostacyclin analogues may also be explored as they have been shown to improve endothelial dysfunction associated to insulin resistance and reduce inflammatory cytokines and hepatic steatosis [49,50].

In summary, the present study provides evidence that endothelial dysfunction observed in NAFLD is at least in part due to COX activity, although thromboxane prostanoid receptor activation does not seem to play a relevant role. In addition, oxidative stress contributes to endothelial dysfunction and improves after antioxidant treatment in this model of NAFLD. This occurs early in the course of the disease, suggesting that both pathways are involved in the pathophysiology of NAFLD and therefore worth exploring as therapeutic targets at early stages of the disease.
